# Influence of Ionic Liquid and Light Intensity on a Polymer Nanostructure Formed in Lyotropic Liquid Crystalline Templates

**DOI:** 10.1021/acsapm.3c01812

**Published:** 2024-02-28

**Authors:** Alexandros Kotsiras, John Whitley, Esmeralda Orozco, C. Allan Guymon

**Affiliations:** Department of Chemical and Biochemical Engineering, University of Iowa, Iowa City, Iowa 52242, United States

**Keywords:** lyotropic liquid crystal, ionic liquid, template, photopolymerization, nanostructure

## Abstract

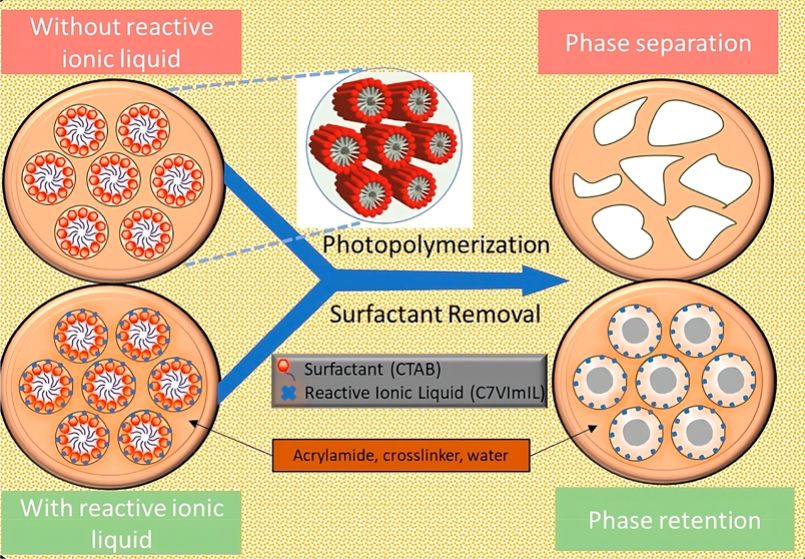

Utilizing self-assembled lyotropic liquid crystal (LLC) templates with radical photopolymerization shows promise in controlling polymer structure on the nanometer scale This control of nanostructure allows tailoring and enhancement of material properties not attainable in traditional polymerization in applications including hydrogels and stimuli-responsive systems. However, thermodynamically driven phase separation between the polymer and LLC templates often hinders the control of local polymer order and resultant polymer properties. This study investigates an alternative method to control the hydrogel nanostructure and avoid phase separation using imidazolium ionic liquids (ILs) in the LLC template while modulating the light intensity used in photopolymerization. The addition of the IL improves the thermodynamic stability and enhances the polymerization rate in the LLC system. The degree of LLC nanostructure retention is increased by increasing light intensities during polymerization. In addition, intermediate concentrations of cross-linker allow a balance between phase stability and cross-linking to lock in LLC morphology. With enhanced retention, the maximum water uptake is significantly higher compared with isotropic controls. These results demonstrate a method to increase the structure on the nanometer scale of a polymer by combining the addition of ILs with the proper selection of light intensity and cross-link density that allows access to unique hydrogel properties. These templated polymers demonstrate enhanced swelling and a stimuli response that show promise in applications ranging from drug delivery to water remediation.

## Introduction

Hydrogels are extensively used in a wide variety of applications, including drug delivery systems,^1^ contact lenses,^2^ tissue scaffolds,^3^ and separation membranes^4,5^ due to their controllable water content, high porosity/permeability, and biocompatibility. Hydrogel properties are typically controlled through manipulation of cross-link density, concentration of monomer, and functionality.^6^ Cross-link density is critical as elastic modulus, swelling behavior, and transport properties directly depend on the type and degree of hydrogel cross-linking.^7^ At the same time, however, changing a specific property of the hydrogel by using cross-linking density typically leads to simultaneous, potentially undesirable changes to other properties, consequently limiting the design flexibility for advanced applications. Therefore, alternative approaches that enable greater control over a hydrogel’s physical properties are of great interest.

Previous studies have examined alternative methods to ultimately control hydrogel material properties, including directing nanomicro-structure via biotemplating,^8^ nanolithography,^9^ two-photon lithography,^10^ phase separation,^11^ and self-assembly.^12^ Additionally, the incorporation of nanoscale structure has enabled one to tune polymer properties that are dependent on the local order of the polymer network, including mechanical strength,^13^ electrical conductivity,^14^ and molecular transport.^15^ These results have demonstrated that hydrogels containing periodic order on the submicrometer scale can result in materials with enhanced strength, biological response, and permeability without changing the original polymer chemistry.^12^

One up-and-coming method to control polymer structure and develop order on the nanometer scale uses self-assembling lyotropic liquid crystals (LLCs) as templates for polymerization.^16^ LLCs are formed with surfactant molecules consisting of a polar (hydrophilic) head and a nonpolar (hydrophobic) tail that self-assemble into a variety of structures when they are diluted in a polar solvent. By altering surfactant concentration, different mesophases assemble, ranging from spherical micelles at lower surfactant concentrations to hexagonal and lamellar phases at higher concentrations shown in Figure 2.^17^ The type of highly ordered nanometer scale mesophase depends on the surfactant concentration, temperature, pressure, and chemistry of the surfactant and monomers, among other factors. LLCs have shown potential as polymerization platforms that direct polymer networks into unique and highly ordered architectures.^12^ When introduced into LLC systems, monomers segregate based on inherent chemical structure within the polar or nonpolar domains of the surfactant molecules adopting the LLC mesophase morphology. Through polymerization, the LLC template nanostructure may be transferred to the polymer using a variety of monomers.^18^ The templated polymers may exhibit enhanced transport, mechanical, and surface properties compared to their isotropic analogs, making them attractive candidates in biomedical and industrial applications, including hydrogels, drug delivery, and water remediation.^19−22^

While LLC templating has demonstrated significant promise in generating nanostructured polymers with enhanced properties and functionality, the retention of the nanostructure during polymerization reactions can be complicated. The most critical challenge is based on the inherent tendency of these templated polymers to phase separate when polymerization occurs with a significant thermodynamically unfavorable decrease in entropy. Such phase separation results in minimal control of polymer properties that depend on polymer structure.^23^ Numerous factors have been identified that facilitate the retention of the LLC nanostructure during radical polymerization, with polymerization kinetics playing a prominent role. For example, initiation rate, monomer segregation behavior, and photoinitiator mobility relate to transferring the LLC template structure to the final polymer.^24−28^ Utilizing photopolymerization has shown particular promise for LLC templating due to inherently rapid initiation rates and temperature independence. These fast rates and the ability to polymerize at lower temperatures may allow the structure of the LLC templates to be kinetically trapped to a much greater degree than other slower initiation methods that require higher temperatures resulting in less ordered structures with larger feature sizes.^29^ Additionally, previous studies have revealed that the photopolymerization of surfactants functionalized with reactive groups can reduce the likelihood of phase separation and achieve higher nanostructure retention.^21^ Other work has shown that adding small amounts of reactive surfactant enhances the stability of the template through polymerization in contrast to systems polymerized with only nonreactive surfactants that are more likely to phase separate.^27,30^

One potential alternative to further enhance structure retention and property control is combining ionic liquids (ILs) and surfactants as contemplated due to potentially synergistic solid interactions.^31−33^ ILs constitute a unique class of primarily organic and ionic materials with a melting point below 100 °C.^34^ ILs may also self-organize into polar and nonpolar regions similar to more traditional surfactants.^35−38^ This self-organization has been found to enhance the polymerization rate due to the local increase of polymerizable molecules.^33,39−41^ Previous studies have shown that numerous factors, including the interaction of template and precursor and chain length, limit this intrinsic self-organization.^31,42^ Recently, a long chain imidazolium IL has been used as a templating agent to create vertically aligned pores in silica films utilizing electrochemically assisted self-assembly.^43^ An alternative method to change the polymerization behavior of IL systems is through the coordination of polymerizable ligands. Such coordination of anions in ILs can induce increased polymerization rate and reactive group conversion.^44^ In addition, combining the greater thermodynamic stability from reactive ILs with LLC templating using photopolymerization could synergize the final polymer nanostructure.

In this study, the nanostructure of polyacrylamide hydrogels is modified through radical photopolymerization using a nonreactive surfactant in concert with a reactive IL. The impact of light intensity on the retention of the nanostructure is examined to identify the effect of faster polymerization kinetics on the retention of the nanostructure with IL/LLC templates. Water swelling of templated materials polymerized at different light intensities is also investigated to determine the effect of changes in the nanometer order on polymer properties. Finally, the influence of cross-linker concentration is correlated with the retention of nanostructure during polymerization upon the addition of the IL. Structure evolution and the swelling behavior are examined with varying cross-link density. Incorporating low IL concentrations in combination with changes in polymerization light intensity may allow effective control of the hydrogel nanostructure and lead to the formulation of materials with unique properties that could be applied in diverse and advanced applications.

## Experimental Section

### Materials

**Figure 1:**
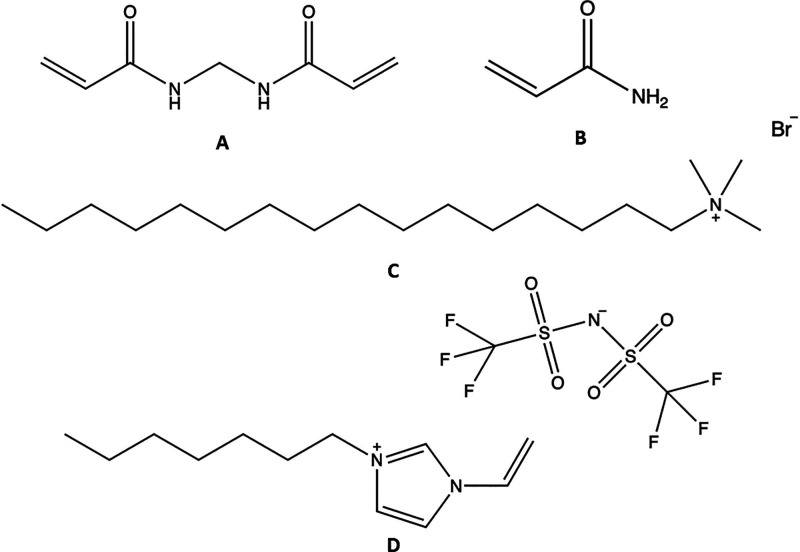
Chemical structures of the monomers and surfactants were used in this study. Shown are (A) *N*,*N*′-methylenebis(acrylamide), (B) acrylamide, (C) hexadecyltrimethylammonium bromide (CTAB) surfactant, and (D) 1-vinyl-3-heptylimidazolium bistriflimide (C7VImIL).

The IL 1-vinyl-3-heptylimidazolium bistriflimide (C7VImIL) monomer was synthesized by reacting 1-vinylimidazole (Sigma-Aldrich) with *n*-bromoheptane (Sigma-Aldrich) according to a method described elsewhere.^44,45^ After the reaction was complete, the bromide ion was exchanged by using lithium bistriflimide to obtain 1-vinyl-3-heptylimidazolium bistriflimide. The monomers used in this work included acrylamide and *N*,*N′*-methylenebis(acrylamide) (Sigma-Aldrich). Additionally, hexadecyltrimethylammonium bromide (CTAB, Sigma-Aldrich) was used as a surfactant. To initiate polymerization, 2,2-dimethoxy-1,2-diphenylethan-1-one (DMPA, Ciba Specialty Chemicals) was used as a radical photoinitiator. The chemical structures of the monomers and surfactants used in this study are shown in Figure 1. All chemicals were used as received.

**Figure 2:**
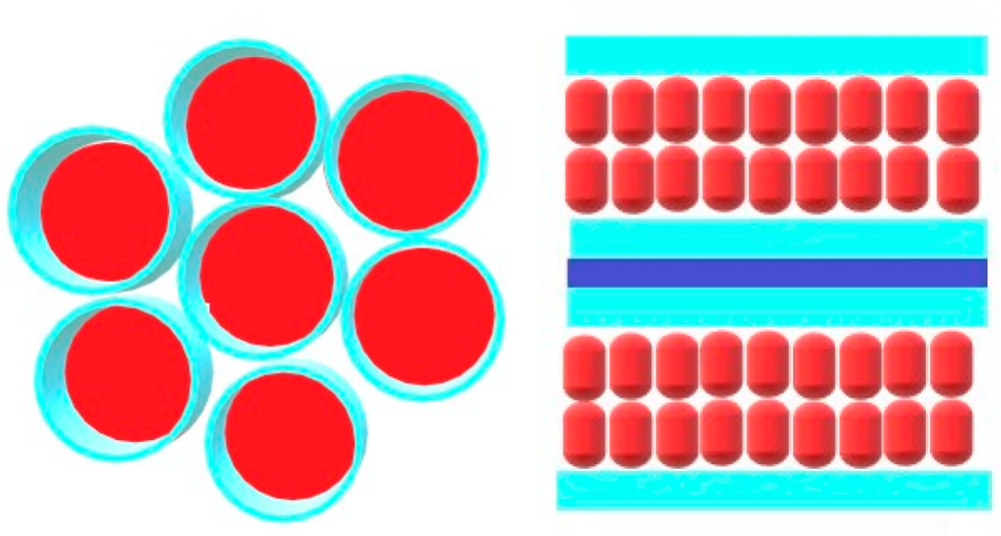
Representation of hexagonal and lamellar LLC mesophases. Polar monomer and cross-linker will be around the water on the polar domain shown in light blue. Hydrophobic tails that form nonpolar domains are shown in red.

LLC solutions were prepared by mixing monomers, surfactants, deionized water, and a photoinitiator. Solution homogeneity was achieved by employing heat, centrifugation, vortex mixing, and mechanical agitation. All samples were prepared using 20 wt % acrylamide, with an acrylamide/*N*,*N*′-methylenebis(acrylamide) mass ratio of 9:1 unless otherwise noted and 1 wt % DMPA with respect to total monomer mass. The LLC-templated samples contained 50 wt % of a combination of CTAB and C7VImIL. As a control, samples with only CTAB and water at a ratio of 5:3, the same ratio of surfactant to water in the polymerizable system, were also examined. Isotropic samples were formulated by incorporating the monomer and IL at the same concentrations in water without any additional surfactant. The polymer samples were prepared by pipetting samples into borosilicate molds (15 mm diameter, 22 mm height), purging with nitrogen for 10 min, and irradiating with the full spectrum of a medium-pressure UV arc lamp (Ace Glass) at an intensity of 10–20 mW/cm^2^ for 10 min from both top and bottom to ensure maximum conversion.

The polymer samples were subjected to solvent exchange with glacial acetic acid for 48 h, with the solvent being replaced periodically to facilitate unreacted monomer, photoinitiator, and surfactant removal. The samples were then dried under vacuum for 48 h.^46^ By comparing sample mass immediately after polymerization to the mass obtained after solvent exchange and drying, at least 95% of the CTAB surfactant was removed with a typical removal over 98%.

### Structural Characterization

The optical anisotropy of the LLC solutions and photopolymerized samples was characterized by using a polarized light microscope (PLM, Nikon, Eclipse E600W Pol) equipped with a hot stage (Instec, Boulder, CO). Before polymerization, images were obtained after heating and slowly cooling to room temperature. The samples were then irradiated and polymerized by using the UV light source as described above. Characteristic birefringent light patterns before and after polymerization were used to indicate ordered phases in the LLC samples both before and after polymerization.^17^

The LLC nanostructure was also characterized through a Nonius FR590 small-angle X-ray scattering (SAXS) apparatus using a standard copper target Röntgen tube with a Ni-filtered Cu Kα line of 1.54 Å as the radiation source, a collimation system of the Kratky type, and a PSD 50 M position sensitive linear detector (Hecus M. Braun, Graz). The specific LLC mesophase was indexed by measuring the ratios of d-spacing from the reflections in the corresponding sample profile. The scattering vector, *q*, was calculated from the angle of the scattered radiation and the X-ray wavelength. The combination of PLM images and SAXS scattering was utilized to determine the LLC/IL formulations’ nanostructure pre- and postphotopolymerization.

### Photopolymerization Kinetics

Polymerization kinetics were investigated by using a PerkinElmer differential scanning calorimeter (photo-DSC) equipped with a high-pressure mercury arc lamp (Omnicure S1500 spot cure system) to initiate polymerization. A 365 nm wavelength filter was used to control the emission spectrum. Samples were prepared by placing approximately 2 mg of solution into crimped aluminum pans covered by transparent fluorinated ethylene propylene copolymer (Teflon FEP, DuPont) film to minimize heat effects caused by water evaporation. Samples were purged with nitrogen for 5 min prior to polymerization to suppress the oxygen inhibition. During the experiments, isothermal conditions were maintained at 30 °C by using a refrigerated circulating chiller, and the heat flow was monitored in real time. The polymerization profiles were compared by normalizing the polymerization heat per unit mass of reactive species during the polymerization.

### Network Swelling

To examine the swelling properties of the polymer, disks were prepared by placing prepolymer formulations into borosilicate molds (15 mm diameter, 2 mm height). Samples were purged with N_2_ for 8 min and photocured using a high-pressure mercury arc lamp (Omnicure S1500 spot cure system) for 10 min at different light intensities, varied from 10 to 20 mW/cm^2^. Polymer swelling was investigated gravimetrically by recording the mass of dried samples and consequently immersing them into excess deionized water maintained at 37 °C. Before the measurement of mass, sample surfaces were patted dry with damp filter paper to remove surface water. Water uptake, i.e., the weight percentage of water in the swollen hydrogel, was calculated using eq 1:
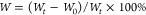
1*W*_*t*_ is the hydrogel’s mass at time *t*, and *W*_0_ is the mass of the dry polymer after a solvent exchange with acetic acid.

## Results and Discussion

Previous studies have shown that utilizing templated LLC mesophases to control polymer nanostructure enables the development of polymers with unique characteristics compared to their isotropic counterparts.^47^ Photopolymerization in and of these ordered systems may enable the formation of polymers with enhanced properties, including water uptake, mechanical stability, and stimuli response. On the other hand, it is often challenging to retain the original LLC nanostructure during the polymerization due to thermodynamically driven phase separation.

This research study investigates a new method to control the nanostructure of LLC-templated polymers during photopolymerization by incorporating polymerizable ionic liquids (ILs) into the polymer network. We hypothesize that copolymerization of monomers with an amphiphilic and compatible polymerizable IL will enhance the interaction between monomers and surfactant and consequently improve the thermodynamic stability within the LLC phase during polymerization. In fact, recent work has shown that the combination of nonreactive and polymerizable surfactants can effectively control polymer nanostructure.^29^ Based on these results, we believe that the IL ion pair may enhance favorable interactions between LLC surfactant and monomers, reducing the thermodynamic instability during polymerization and, thus, the accompanying driving forces that induce phase separation.

### IL Concentration Impact on Nanostructure Retention

**Figure 3:**
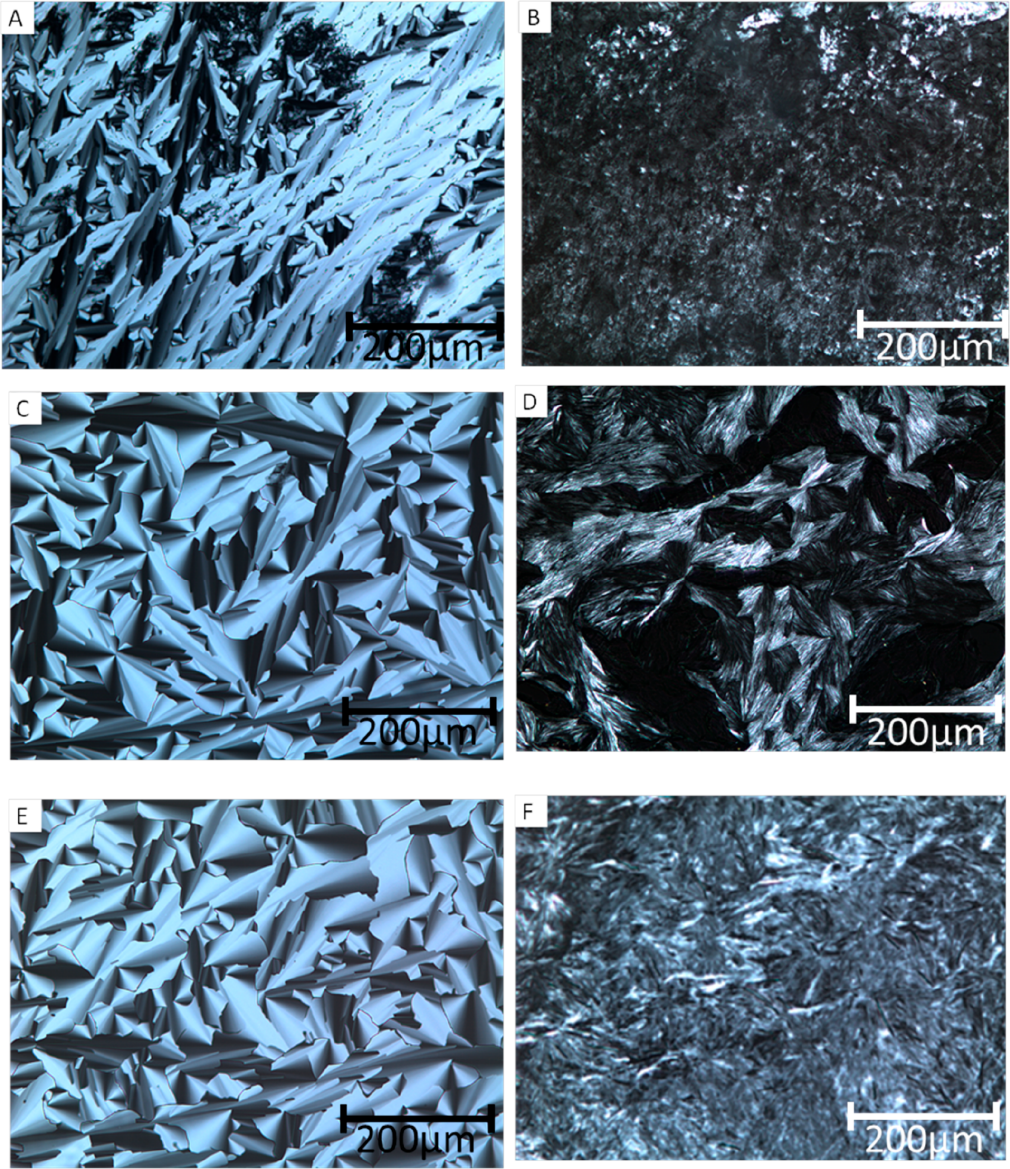
Polarized light micrographs (100×) of 20 wt % cross-linked acrylamide templated with different ratios of CTAB and C7VImIL surfactants (50 wt % total). Shown are systems with 0 wt % C7VImIL (A) before and (B) after photopolymerization, 10 wt % C7VImIL (C) before and (D) after photopolymerization, and 15 wt % C7VImI (E) before and (F) after photopolymerization.

To determine the impact of IL on LLC mesophase formation and retention, acrylamide/CTAB systems containing various amounts of ILs were examined by using PLM. Figure 3 shows the PLM images of templated acrylamide with different ratios of CTAB and C7VImIL before and after polymerization. Samples containing 0, 10, and 15 wt % of C7VImIL before polymerization (Figure 3A,C,E) show the characteristic birefringence and fan-like optical texture of a hexagonal LLC nanostructure. After polymerization at 10 mW/cm^2^, the templated polyacrylamide systems display less defined optical textures (Figure 3B,D,F), implying that the original LLC phase is changed to some extent during polymerization. However, as the amount of C7VImIL in the mixture increases, the conservation of the optical texture after polymerization is enhanced, suggesting that sufficient amounts of IL incorporated into the LLC template do enhance nanostructure retention to at least some degree during polymerization.

**Figure 4:**
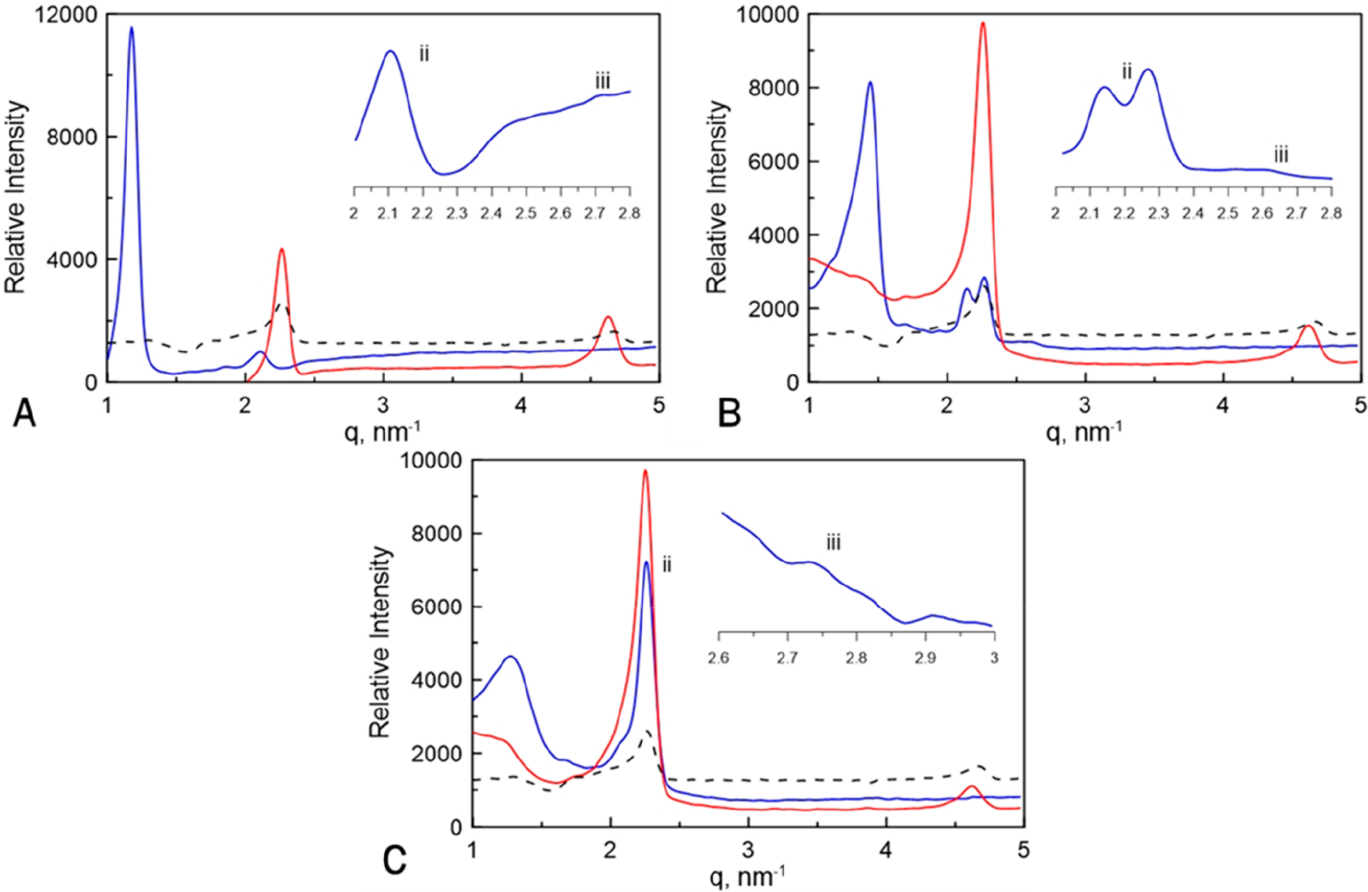
SAXS scattering profiles before and after photopolymerization of samples templated with varying concentrations between nonreactive CTAB and polymerizable C7VImIL surfactants polymerized at 10 mW/cm^2^. Shown are profiles of samples with only 5:3 weight ratio CTAB:water (black dashed line) and before (blue solid line) and after (red solid line) polymerization of (A) 0/50 wt % C7VImIL/CTAB, (B) 10/40 wt % C7VImIL/CTAB, and (C) 15/35 wt % C7VImIL/CTAB. Insets are also shown to indicate the secondary reflections that identify the mixed biphasic nanostructure.

PLM results were corroborated by studying the effect of the IL concentration on the LLC structure of the samples before and after polymerization via SAXS. The direct comparison between these two methods allows the determination of the type of LLC mesophase and, simultaneously, the degree of nanostructure retention after polymerization. Figure 4 shows SAXS profiles before and after polymerization at 10 mW/cm^2^ for the mesophases formed from 20 wt % acrylamide templated with 50 wt % surfactant at different ratios of CTAB and C7VImIL in water.

The scattering profile observed before the photopolymerization of acrylamide templated with 50 wt % CTAB (Figure 4A) exhibits a characteristic peak ratio of an LLC hexagonal and lamellar mesophase mixture. The SAXS profile after polymerization exhibits large changes in the position of scattering maxima, and scattering intensity is significantly reduced compared to the SAXS profile obtained before polymerization. These changes between the SAXS profiles indicate that the original LLC structure is not retained during the polymerization of acrylamide templated with CTAB. Similar trends can be observed by increasing the concentration of C7VImIL to 10 wt % and reducing CTAB concentration to 40 wt % (Figure 4B). The significant changes of SAXS profiles before and after polymerization for the samples that contain 0 and 10 wt % of C7VImIL, along with PLM results, indicate that the original nanostructure is not retained and the polymer has phase separated to a large degree during polymerization. Additionally, the degree of phase separation is further highlighted by comparing the scattering of these cured samples with the scattering of a control formulation containing only CTAB and water in the same ratio. This formulation represents the behavior of the surfactant/water system if the polymer completely phase separates with no interaction with the LLC phase. The peak positions of the cured samples and the CTAB/water system are the same, supporting the idea that the LLC template no longer interacts with the polymer network and has phase separated.

The SAXS profiles for the samples incorporating 15 wt % C7VImIL before polymerization exhibit a diffraction peak ratio indicative of biphasic LLC morphology. However, in this case, the mesophase does not appear to be disrupted to nearly the same degree through photopolymerization.

The postpolymerization profile indicates greater retention of the nanostructure with a strong primary reflection with increased peak intensity as compared to the prepolymerization profile. The peak positions before and after polymerization are similar, suggesting a very similar phase structure.

### Light Intensity Effects

**Figure 5:**
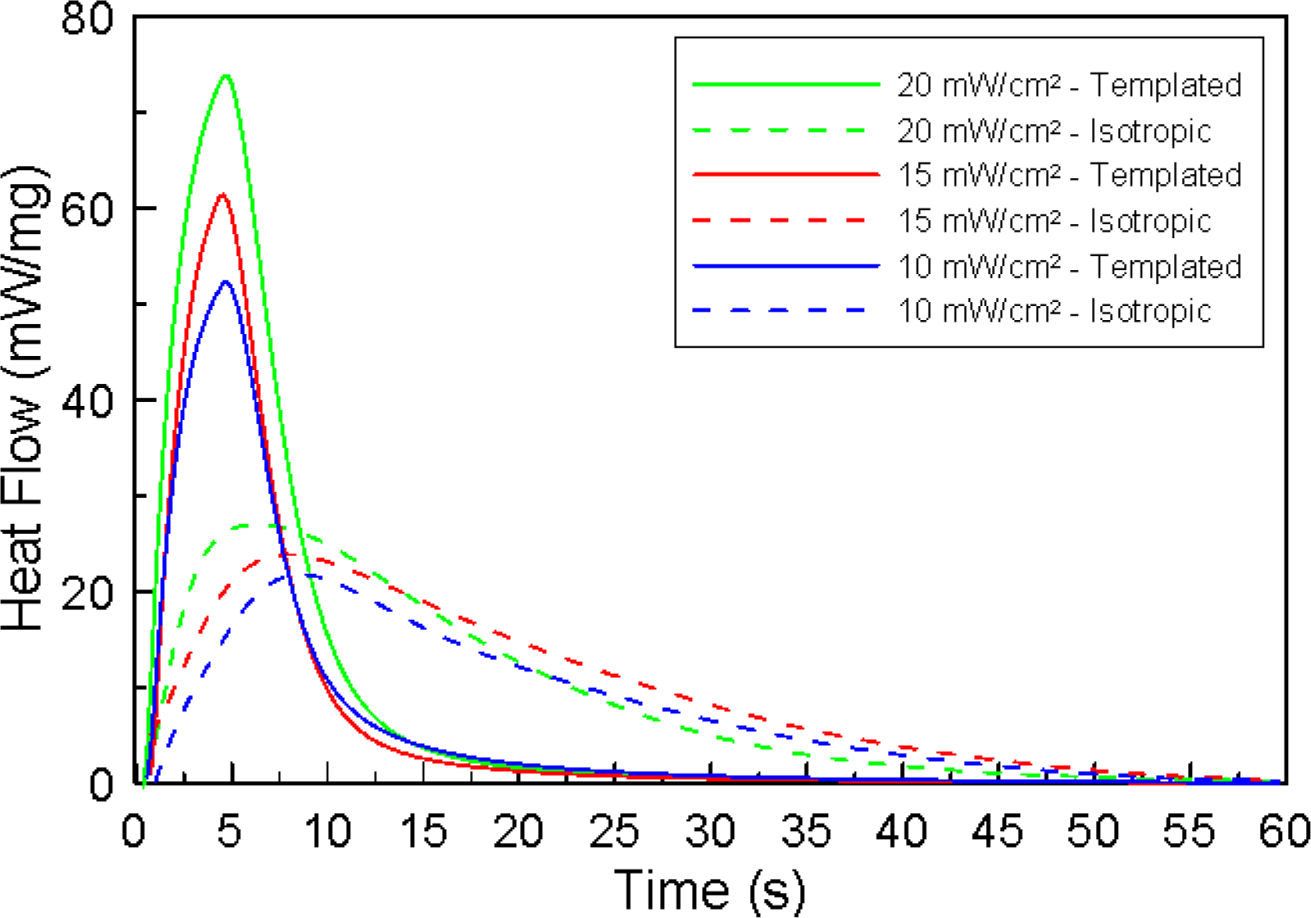
Normalized polymerization heat flow as a function of time for 20/30 wt % C7ImIL/CTAB polyacrylamide photopolymerized using varying photopolymerization light intensities. Shown are templated polyacrylamide polymerized at 10 (blue solid line), 15 (red solid line) and 20 (green solid line) mW/cm^2^ as compared to their isotropic analogues at 10 (blue dashed line), 15 (red dashed line) and 20 (green dashed line) mW/cm^2^.

Figure 4 shows that increasing the amount of IL incorporated into the network results in an enhanced retention of the nanostructure. Prior work has also shown that light intensity can significantly affect the retention of the nanostructure.^44^ The combined effect of light intensity with the addition of IL was examined to determine if more effective control of nanostructure can be achieved. The polymer’s local order directly affects photopolymerization kinetic behavior in LLC systems.^29^ Prior work has shown that higher degrees of LLC mesophase retention are often accompanied by significant increases in polymerization rate. To determine if the polymer nanostructure and the degree of retention affect the polymerization rate, the polymerization kinetic behavior of the LLC/IL templated systems was examined. Figure 5 shows the photopolymerization kinetic profiles for both LLC-templated and isotropic acrylamide systems polymerized with different light intensities. Isotropic solutions polymerize relatively slowly with different light intensities compared to the corresponding templated samples. Additionally, the isotropic samples exhibit an increase in heat flow due to light intensity, as would be expected. In fact, an increase of about 35% is observed between the two limiting light intensities examined. This increase is associated with the increase in initiating radical concentration caused by the increase in light intensity.

On the other hand, the heat flow released during polymerization for LLC-templated systems increases dramatically when compared to that of isotropic counterparts. More specifically, the maximum heat flow for samples polymerized at 10 mW/cm^2^ doubles with the addition of templating surfactants. Similar trends are observed at the other light intensities, with 20 mW/cm^2^ showing the highest rate increase with approximately three times the maximum heat release compared with isotropic counterparts. Interestingly, the maximum heat flow of polymerization for the templated samples appears to be connected to enhanced LLC structure retention. The maximum heat flow increase between the two limiting light intensities is approximately 55%. The observed increase of 20% enhancement compared to that shown for the isotropic samples may be due to an increased local concentration of reactive species due to segregation in the LLC mesophase at later stages of the polymerization reaction. These findings suggest that higher light intensity may result in greater retention of the nanostructure.^12^

**Figure 6:**
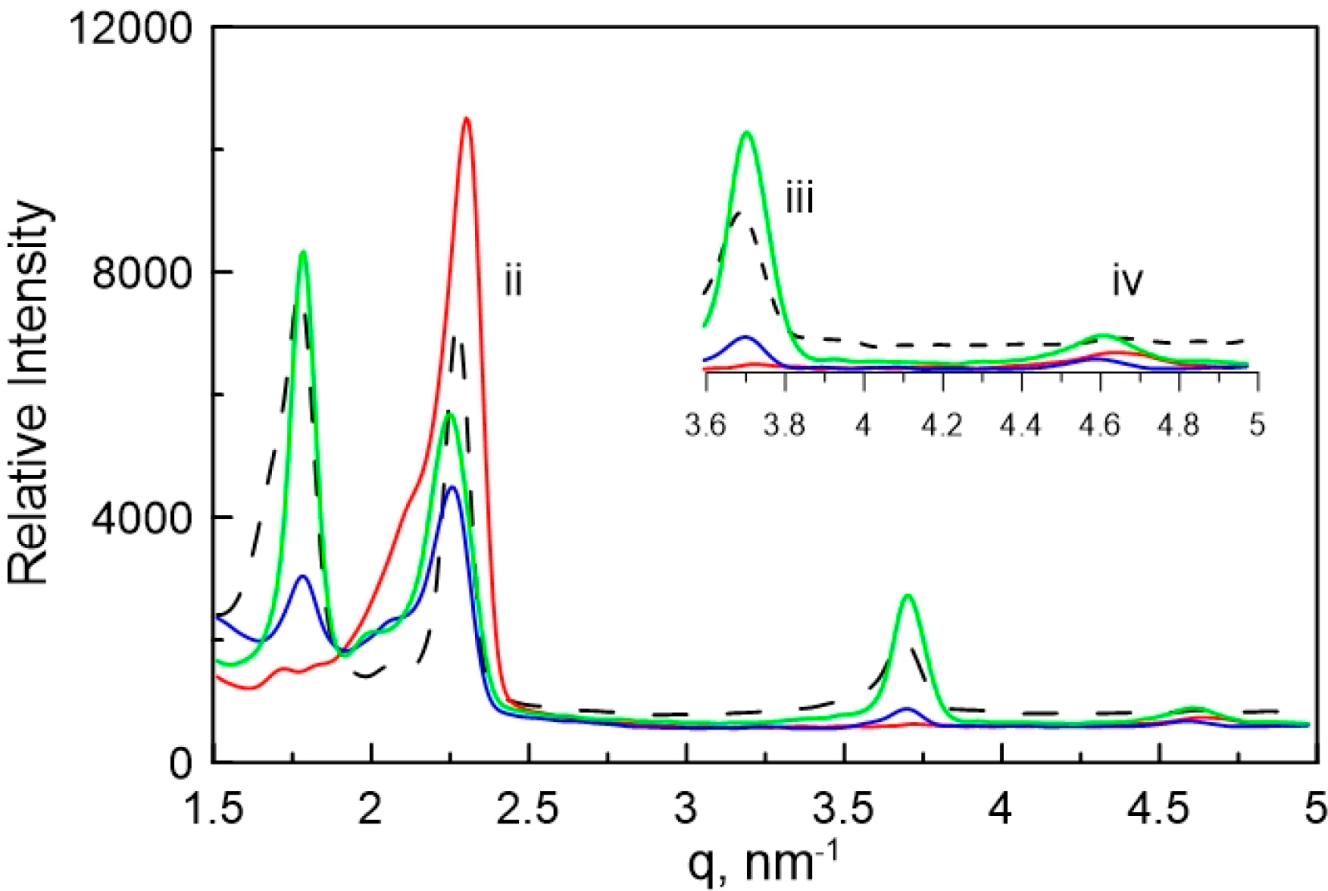
SAXS scattering profiles of 20/30 wt % C7VImIL/CTAB before photopolymerization (black dashed line) and after photopolymerization at 10 mW/cm^2^ (red solid line), 15 mW/cm^2^ (blue solid line), and 20 mW/cm^2^ (green solid line). Insets are also shown to indicate the secondary reflections that identify the hexagonal and lamellar mesophase mixture.

To understand how the light intensity and degree of retention are related, as implied by the kinetics data, the nanostructure of the samples was further examined using SAXS, comparing the scattering profiles cured at different light intensities. By examination of the corresponding SAXS profiles (Figure 6), a direct relationship between nanostructure retention and light intensity was observed. The scattering profile before polymerization corresponds to a mixed hexagonal and lamellar phase. Photopolymerization with 10 mW/cm^2^ induces changes to the position of the peak maxima. These new peak positions correspond to the scattering profile of a formulation with the same ratio of only CTAB and water, implying phase separation of the polymer network. When the light intensity is increased to 15 mW/cm^2^, the SAXS profile is altered significantly. The peak positions at this light intensity are retained through polymerization, although the intensity of both the primary and secondary peaks is reduced considerably. Further increasing the light intensity to 20 mW/cm^2^ shows no shift in *q* with similar primary and secondary peak intensities, implying significant retention of the original nanostructure during photopolymerization.

These results suggest that incorporation of the C7VImIL into the templated polymer network stabilizes thermodynamically the LLC phase and, with sufficiently fast polymerization from higher light intensities, results in enhanced retention of the LLC mesophase.

**Figure 7:**
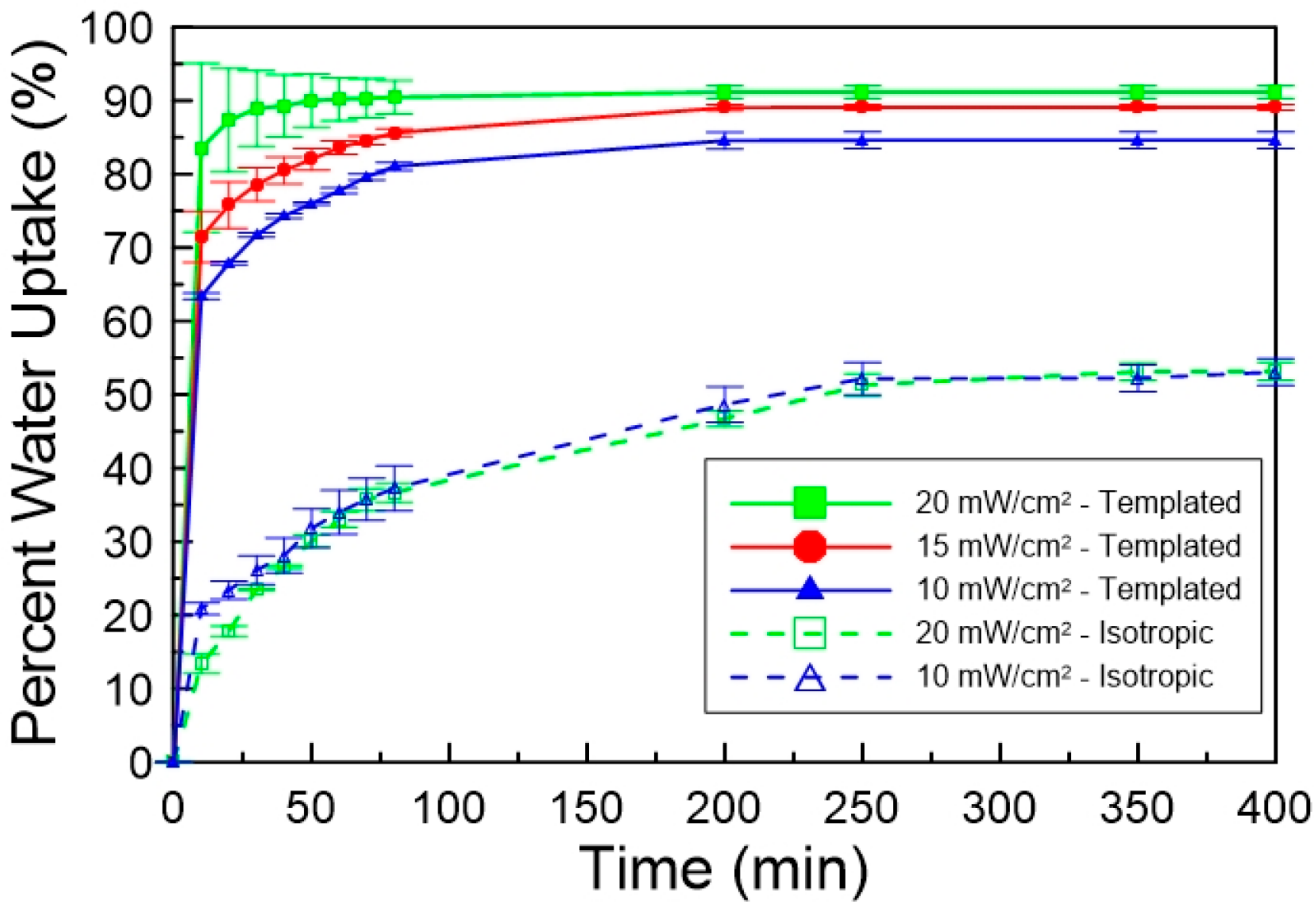
Mass percentage water content as a function of time for polyacrylamide hydrogels photopolymerized by using varying polymerization light intensities. Also included is water uptake of isotropic polyacrylamide controls with the same composition of LLC templated systems with CTAB replaced by additional water. Shown are the templated samples polymerized at 10 (blue solid line), 15 (red solid line), and 20 (green solid line) mW/cm^2^and their respective isotropic controls polymerized at 10 (blue dashed line) and 20 (green dashed line) mW/cm^2^. CTAB surfactant was removed by solvent exchange prior to analysis.

Nanostructure formation in polyacrylamide hydrogel networks results in substantial changes in material properties, transforming their potential utility in applications.^18^ The different degrees of nanostructure retention at these three different light intensities of polymerization may consequently influence polymer properties, including swelling behavior. To determine if the nanostructure changes influence swelling, the water absorption kinetics and maximum water uptake for polyacrylamide gels were examined when the samples were polymerized at different light intensities. Figure 7 shows the percentage water uptake as a function of time for photopolymerized samples at 10, 15, and 20 mW/cm^2^ with 20 wt % C7VImIL. The samples polymerized with 15 and 20 mW/cm^2^ exhibit the greatest swelling uptake, swelling to approximately 90% of their original mass in water, whereas the phase-separated system, polymerized at 10 mW/cm^2^, swells only slightly over 80%. The increase of water uptake of almost 10% by only changing the light intensity of polymerization for the nanostructured samples could indicate significant enhancement in transport properties. Additionally, the templated samples with increased nanostructure retention after polymerization showed much greater rates of water swelling, reaching equilibrium in approximately 80 min, as compared to the phase-separated samples that required at least 150 min to reach equilibrium. This rate increase can be attributed to more well-defined pores on the nanoscale that allow more effective transport. The effect of nanostructure retention is even more dramatic in comparison to respective isotropic controls. Water uptake for the isotropic samples remained only around 50%, with similar swelling kinetics for all light intensities.

### Cross-Linker Concentration

Cross-linking also significantly affects the retention of the LLC nanostructure through polymerization. The number of cross-links can significantly affect the kinetic behavior of the system as cross-link density impacts the rate and the ultimate conversion of double bonds due to diffusion limitations during the reaction.^48,49^

**Figure 8:**
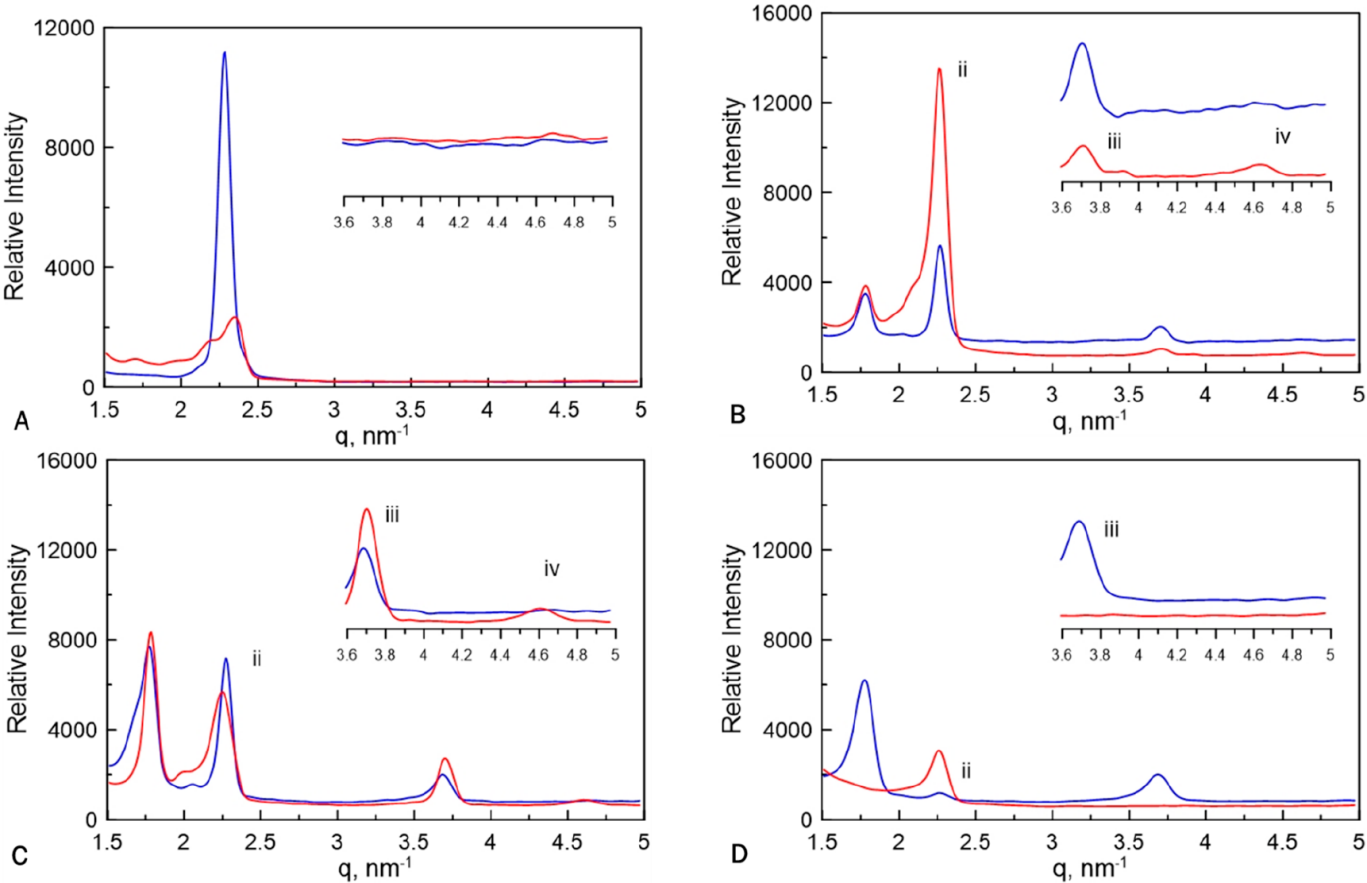
SAXS scattering profiles before and after photopolymerization of systems templated with varying concentrations of cross-linker with respect to the monomer mass. All the examined samples are templated with a ratio of 30/20 wt % C7ImIL/CTAB. Shown are profiles of samples before (blue solid line) and after polymerization (red solid line) in (A) 0 wt % cross-linker, (B) 0.50 wt % cross-linker, (C) 1.00 wt % cross-linker, and (D) 2.00 wt % cross-linker. Insets indicate the secondary reflections that identify the degree of phase retention.

Additionally, cross-linking is necessary to enable the kinetic trapping of the polymer nanostructure. On the other hand, cross-linking molecules are typically nonmesogenic, thus decreasing the thermodynamic stability of the LLC phase. Therefore, the effect of the cross-linker amount was examined to determine its impact on the nanostructure retention of LLC systems. SAXS analysis before polymerization (Figure 8) reveals that mixed hexagonal and lamellar mesophases were formed in samples with up to 2 wt % cross-linker, similar to previous results. For the samples without cross-linker, only a strong primary reflection is observed before polymerization at the same position with the scattering of a control formulation containing only CTAB and water. Upon polymerization, a significant reduction in the intensity of the primary peak results. The substantial changes in scattering profiles indicate that the absence of a cross-linker in the LLC mixture results in the loss of the nanostructure during photopolymerization.

Enhanced retention of the nanostructure is observed when adding cross-linker up to 1 wt %. Figure 8B shows the scattering before polymerization with multiple reflections that indicate mixed hexagonal and lamellar mesophases. Upon polymerization, the primary peak positions do not change with enhanced signal, suggesting significant preservation of the nanostructure. These results verify that even small amounts of cross-linker induce significantly increased mesophase morphology retention through polymerization with a higher percent cross-linker (Figure 8C). The intensity and position of the peaks remain similar before and after polymerization, enabling nanostructure retention similar to that with a 0.5 wt % cross-linker. On the other hand, by increasing the concentration of cross-linker to 2 wt % (Figure 8D), the position of the scattering peaks changes after polymerization, suggesting that the structure of the hydrogel has been disrupted. These results reveal that the cross-link density affects the type of phase formed and the stability of the phase before polymerization.

**Figure 9:**
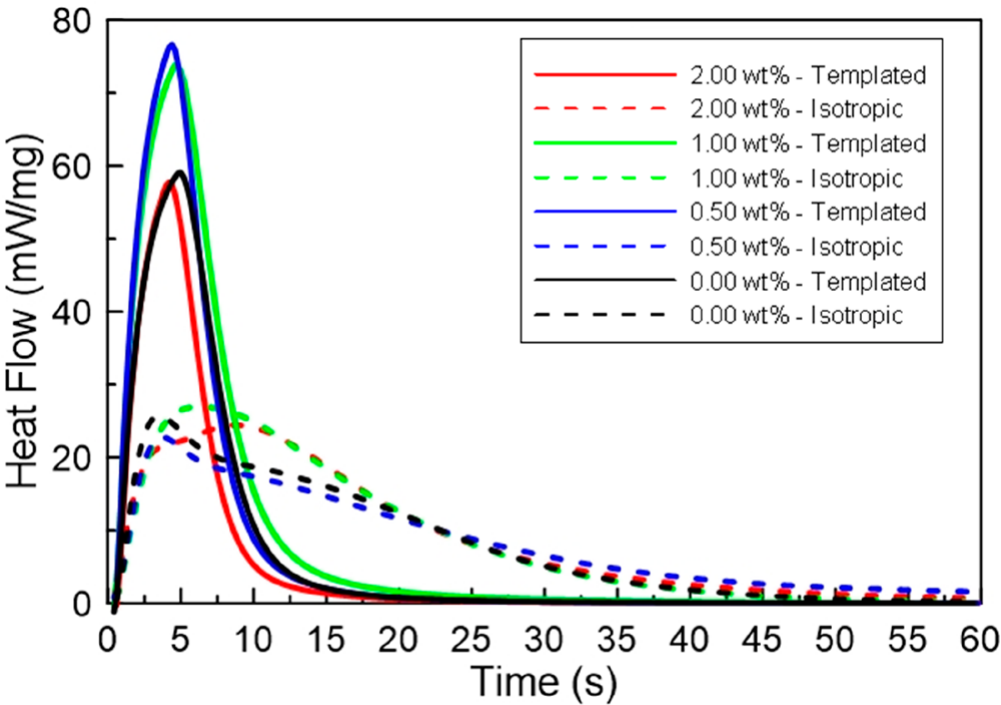
Normalized polymerization heat flow as a function of time by using varying cross-linker concentrations. Shown are polyacrylamide cross-linked samples with 0.00 wt % (black solid line), 0.50 wt % (blue solid line), 1.00 wt % (green solid line) and 2.00 wt % (red solid line) and their isotropic analogues 0.00 wt % (black dashed line), 0.50 wt % (blue dashed line), 1.00 wt % (green dashed line), and 2.00 wt % (red dashed line).

Intermediate concentrations of cross-linkers resulted in the highest degree of nanostructure retention. As mentioned previously, photopolymerization kinetic behavior in LLC systems is affected by the local order and may be used as a means to understand the evolution of polymer structure during polymerization. As such, the heat flow of polymerization for different concentrations of cross-linker in solution as a function of time was examined (Figure 9). The isotropic controls display relatively slow polymerization kinetics. Higher amounts of cross-linker result in slightly greater heat release and polymerization rates. On the other hand, the heat flow during polymerization is notably influenced by the addition of a cross-linker for the templated samples. All LLC-templated systems with a cross-linker show enhanced heat flow of polymerization compared to the isotropic control. Adding 0.5 or 1 wt % cross-linker not only enhances the maximum heat flow of polymerization by a factor of 3 compared to isotropic controls but also decreases the time to reach the maximum conversion by a factor of 4. The addition of a cross-linker to the templated system increases the maximum heat flow by about 30% when comparing the samples of intermediate cross-linker amounts with the samples with no cross-linker, a much greater change than that observed when increasing the cross-linker in the isotropic systems. A further increase in cross-linker concentration exhibits lower polymerization rates, similar to that observed for the systems without cross-linker. These changes in kinetic behavior agree with the SAXS profiles that showed disruption of the phase at great amounts of cross-linker, confirming that the polymerization rate is greatly affected by the retention of LLC nanostructure. In fact, systems with a high degree of LLC template nanostructure frequently exhibit significantly faster photopolymerization rates.^26^

**Figure 10:**
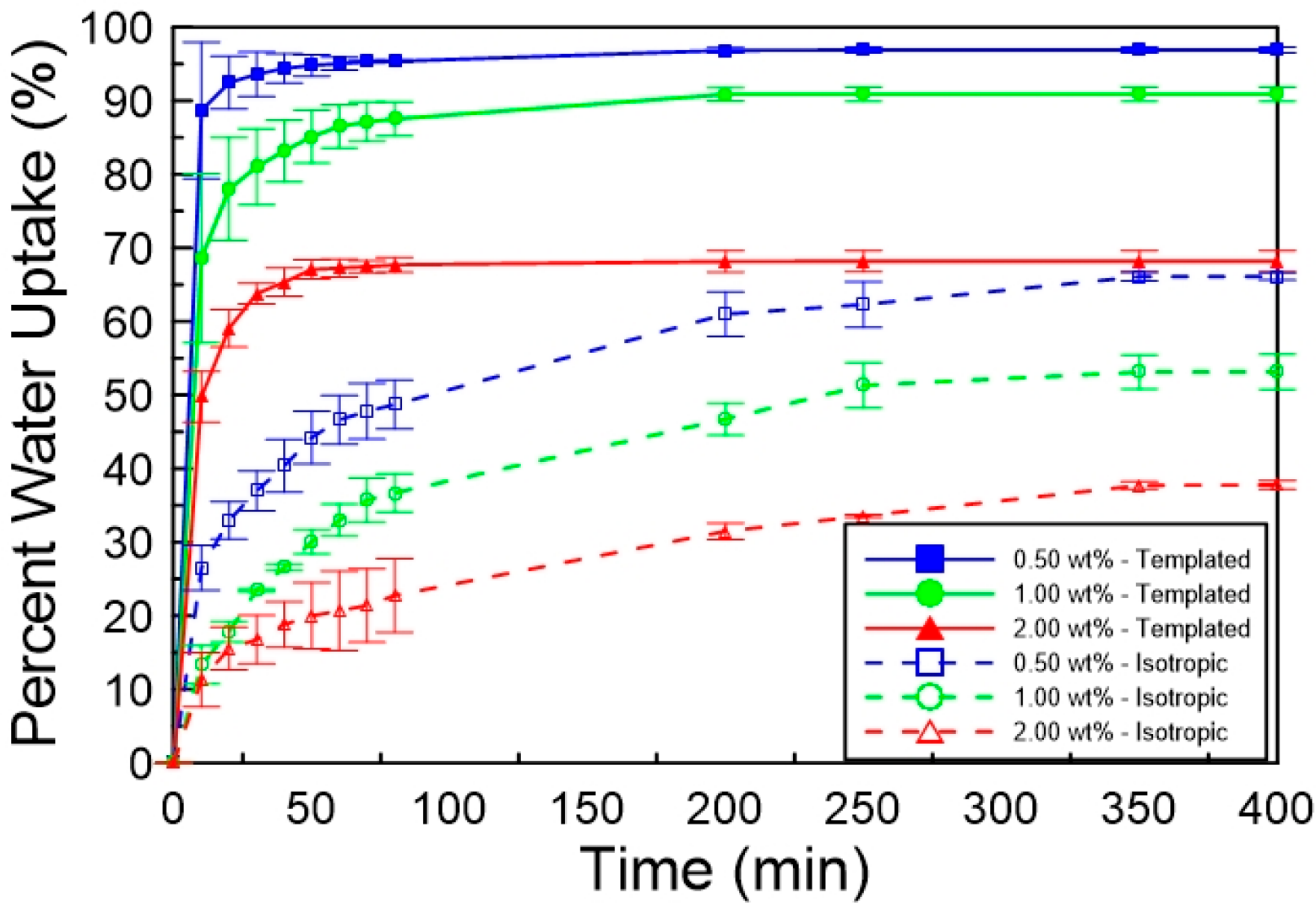
Water percentage uptake as a function of time of polyacrylamide hydrogels photopolymerized cross-linked. Shown are the templated samples cross-linked with 0.50 wt % (blue solid line), 1.00 wt % (green solid line), and 2.00 wt % (red solid line) of *N*,*N*′-methylenebis(acrylamide) and their respective isotropic controls with 0.50 wt % (blue dashed line), 1.00 wt % (green dashed line), and 2.00 wt % (red dashed line). The CTAB surfactant was removed by solvent exchange prior to analysis.

These changes in morphology may induce differences in the transport properties of the resulting polyacrylamide hydrogel samples, as noted above, with increased light intensity. Water swelling was studied to examine the effects of cross-link concentration on polymer properties. Figure 10 shows the mass percentage of water uptake as a function of time at different cross-linker concentrations. The absence of cross-linker produced materials that break easily and do not remain intact during water uptake, and thus swelling could not be measured for the samples with 0 wt % cross-linker. The templated samples exhibit the fastest swelling kinetics, reaching equilibrium in 200 min for all three tested cross-linker concentrations. Corresponding isotropic controls require an additional 150 min to reach equilibrium, and the slope of the swelling kinetics indicates a much slower swelling uptake. Furthermore, templated samples with 0.5 and 1 wt % cross-linker swell to a much greater degree, taking up more than 90% of their original mass in water. On the other hand, the templated samples with 2 wt % cross-linker exhibit a reduction of about 25% in maximum swelling. While a decrease is expected with increased cross-link density, the significantly lower degree of swelling for the samples with the highest cross-linker concentration is also likely due to the network phase-separating to some degree, producing a system with significantly less order with a decreased extent of swelling. The nanostructure may influence diffusion in the hydrogel with the direct transport pathways provided by the LLC mesophase nanopores.^30^ Isotropic controls showed significantly reduced maximum swelling compared to the templated samples. Additionally, the maximum swelling decreased as the concentration of cross-linker increased. Interestingly, the most significant change between the swelling of templated and isotropic controls is observed in the hydrogel cross-linked with 1 wt %, in which an increase in swelling of about 40% can be achieved with templated local order in the final polymer, suggesting that controlling polymer nanostructure plays a critical role on the final polymer properties.

From PLM, SAXS, and polymerization kinetics data, it is evident that incorporating ILs in conjunction with higher radical photopolymerization light intensities enhances the thermodynamic stability of the LLC mesophase. When appropriate degrees of cross-linking are used, the degree of template structure transferred to the final polymer network during polymerization is enhanced significantly. In addition, water uptake studies indicate that the retention of nanostructure elicits significant changes, doubling the degree of swelling uptake relative to polymers with the same chemical composition but a nonordered network architecture.

## Conclusions

Control of the hydrogel nanostructure through photopolymerization with LLC templates has been achieved by the addition of an imidazolium IL (C7VImIL). Adding IL significantly increases the degree of the LLC nanostructure transferred to polyacrylamide hydrogels during polymerization. Nanostructure retention is enhanced with an increased concentration of IL. Additionally, increasing the photopolymerization light intensity significantly increases the degree of retention of LLC mesophase order templated within the final polymer during polymerization. The higher light intensities produce more initiating radicals, resulting in a faster polymerization rate that enables better kinetic trapping of the structure. Cross-link density also significantly affects the final polymer order, with intermediate concentrations of cross-linker, resulting in successful retention of the nanostructure during polymerization. This structure evolution also impacts polymerization kinetics, with rates enhanced up to three times with templated systems compared to both phase-separated and isotropic controls. Physical properties are also affected, with templated polyacrylamide hydrogels showing doubled swelling in water and 150% faster swelling rates relative to those of their isotropic counterparts. The ability to effectively tailor polymer structure upon the addition of reactive IL, when combined with only slight changes in polymerization light intensity and cross-link density, allows retention of the LLC nanostructure through radical polymerization within LLC templates. These results show promise for the development of hydrogels with well-organized nanostructures and enhanced transport properties that could provide direct opportunities in applications ranging from drug delivery to water remediation.
